# Fewer tumour draining sentinel nodes in patients with progressing muscle invasive bladder cancer, after neoadjuvant chemotherapy and radical cystectomy

**DOI:** 10.1007/s00345-019-03025-w

**Published:** 2019-11-23

**Authors:** Julia Alvaeus, Robert Rosenblatt, Markus Johansson, Farhood Alamdari, Tomasz Jakubczyk, Benny Holmström, Tammer Hemdan, Ylva Huge, Firas Aljabery, Susanne Gabrielsson, Katrine Riklund, Ola Winqvist, Amir Sherif

**Affiliations:** 1 Department of Surgical and Perioperative Sciences, Urology and Andrology, Umeå University, 901 85 Umeå, Sweden; 2 Department of UrologyKarolinska Institutet, Stockholm South General Hospital, Stockholm, Sweden; 3 Department of Urology, Sundsvall Hospital, Sundsvall, Sweden; 4 Department of Urology, Västmanland Hospital, Västerås, Sweden; 5 Department of Urology, Länssjukhuset Ryhov, Jönköping, Sweden; 6 Department of Surgical Sciences, Uppsala University, Uppsala, Sweden; 7 Division of Urology, Department of Clinical and Experimental Medicine, Linköping University, Linköping, Sweden; 8 Division of Immunology and Allergy, Department of Medicine Solna, Karolinska Institutet, Stockholm, Sweden; 9 Department of Radiation Sciences, Umeå University, Umeå, Sweden; 10 Department of Clinical Immunology, Karolinska University Hospital, Stockholm, Sweden

**Keywords:** Urinary bladder neoplasms, Neoadjuvant therapy, Cisplatin, Sentinel lymph node biopsy, Cystectomy

## Abstract

**Purpose:**

To examine the relationship between the number of tumour draining sentinel nodes (SNs) and pathoanatomical outcomes, in muscle-invasive bladder cancer (MIBC), in patients undergoing neoadjuvant chemotherapy (NAC) and radical cystectomy (RC).

**Materials and Methods:**

In an ongoing prospective multicenter study, we included 230 patients with suspected urothelial MIBC from ten Swedish urological centers. All underwent TURb and clinical staging. From the cohort, 116 patients with urothelial MIBC; cT2-cT4aN0M0, underwent radical cystectomy (RC) and lymphadenectomy with SN-detection (SNd). 83 patients received cisplatin-based NAC and 33 were NAC-naïve. The number and locations of detected SNs and non-SNs were recorded for each patient. The NAC treated patients were categorized by pathoanatomical outcomes post-RC into three groups: *complete responders (CR), stable disease (SD)* and *progressive disease (PD).* Selected covariates with possible impact on SN-yield were tested in uni -and multivariate analyses for NAC-treated patients only.

**Results:**

In NAC treated patients, the mean number of SNs was significantly higher in CR patients (3.3) and SD patients (3.6) compared with PD patients (1.4) (*p* = 0.034). In a linear multivariate regression model, the number of harvested nodes was the only independent variable that affected the number of SNs (*p* = 0.0004).

**Conclusions:**

The number of tumor-draining SNs in NAC-treated patients was significantly lower in patients with progressive disease.

## Introduction

Urinary bladder cancer (UBC) is the fourth most common malignancy in men and the eighth most common in women, in the Western world [1]. Approximately 25–30% of bladder tumours are muscle-invasive (MIBC) [1, 2]. MIBC is associated with high risk of regional and distant metastatic spread, the latter with a median survival of 15 months albeit maximum oncological treatment [3]. Treatment of localized MIBC (T2a-T4aN0M0) is radical cystectomy (RC) with regional lymphadenectomy (LND). However, despite radical excision, local recurrence or distant metastases develop in around 50% of patients, probably due to early micrometastases [4]. In attempts to eliminate early dissemination, cisplatin-based combination neoadjuvant chemotherapy (NAC) is recommended to all medically fit patients with clinically localized MIBC [5, 6]. NAC is administered systemically in 3–4 cycles pre-RC. NAC is associated with significant overall survival (OS) benefits; a large meta-analysis assigned it to an 8% absolute increase in 5-year OS [7]. Especially good survival benefits have been seen in patients where NAC induces complete downstaging (CD) of the primary tumour, suggesting CD to be a surrogate marker for efficacy on dissemination [8].

A sentinel node (SN) is defined as the primary tumour-draining lymph node (LN) [9] and is considered being the primary site of metastasis. Yet, evidence from recent years of SN-research in MIBC shows that the number of detectable SNs often exceed one single node [10–12]. SN-detection (SNd) can be performed by peritumoral injection of radioactive tracer and intraoperative examination with handheld γ-probe [11–14]. Recently, fluorescence-guided intraoperative imaging of lymphatics, using Indocyanine green (ICG) shows promising results [15].

The SN-concept in MIBC was originally introduced with aims of improving identification of LN-metastases or determining the extent of LN-dissection. However, several studies have shown SN-detection to be of limited or no use in nodal staging [12, 14]. Instead, focus on SNd in MIBC has shifted to its role in immunobiological research [16–21]. Because a SN is the compartment where the host immune system first encounters tumour-derived antigens, it is also a good site for extracting tumour reactive lymphocytes for use in adoptive T-cell immunotherapy [22, 23]. Recent SN-research also shows that NAC promotes antitumor T-cell responses in MIBC, by activating T-effector cells (Teffs) and reducing the immunosuppressive activity of regulatory T-cells (Tregs) in SNs. Higher Teff to activated Treg ratio has been established in patients where NAC has induced CD [21].

What remains unanswered is the relationship between the number of tumour-draining SNs and pathoanatomical responses to NAC. Considering the SN-role in the immune defence against cancer, we speculate that the greater the number of SNs in a patient, the higher the chance of non-progression due to NAC. In 2016 Rosenblatt et al. [14] reported on the feasibility of SN-detection in NAC-patients, regardless of pT-stage. We now investigate the number of SNs and its association to pathoanatomical status after NAC, in an enlarged prospective cohort.

## Materials/patients

**Fig. 1:**
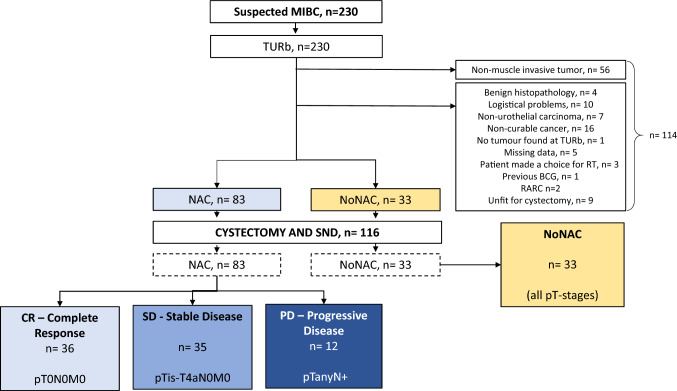
Flow chart of patient inclusions and subgroups. In total, 230 patients were enrolled to undergo TURb for suspected urothelial MIBC. 56 patients were histopathologically diagnosed as non-muscle invasive bladder cancer (NMIBC) and subsequently excluded. 58 patients were excluded due to other reasons, listed in the figure. The remaining patients underwent cystectomy and sentinel node detection (*n* = 116) and was subgrouped according to NAC treatment status. The NAC-patients (*n* = 83) were further stratified into; complete response, CR, (pT0N0M0), stable disease (SD) (pTis-pT4aN0M0), and progressive disease (PD), (pTanyN+ and/or M+)

230 patients with suspected urothelial MIBC from ten Swedish urological centers were included in a non-randomized prospective trial. Enrolment started in May 2013 and closed in December 2018. Main inclusion criterion was suspected urothelial MIBC. Reasons for exclusion included; previous BCG-therapy, non-muscle invasive UBC following TURb and robot-assisted laparoscopic radical cystectomy (RARC). For all exclusion criteria, see flow chart (Fig. 1).

## Methods

SNd by radioactive technetium was performed in a standardized fashion across all RC-centers as previously described [14]. A handheld intraoperative Geiger counter registered radioactivity in suspected lymph nodes, by *counts per minute of ionization events (CPM).* Lymph node detection was performed according to the intended same template: Bilateral Obturator fossae, External Iliac artery bilaterally, Common Iliac artery up to mid-level. Any in situ detected sentinel nodes at any other locations were also included apart from the intended template. Nodal specimens were defined by histopathology as true LNs or not. A true LN, w/wo metastasis, with detected CPM ≥ 10 was defined as a *SN*. If a nodal SN-specimen was found containing > 1 LN in the pathology evaluation, the detected CPM-value was divided by the number of contained nodes. Specimens with CPM > 10 not containing any lymphatic tissue, were *false positive (FP)-*detections. If an undetected specimen showed LN-metastasis, it was named a *false negative (FN)*-detection.

### Statistics

Differences in numerical and ordinal variables were tested using one-way ANOVA. For categorical data, the Chi-squared test was used. Furthermore, selected covariates were analyzed in a multivariate linear model for possible confounders impacting SN-yield. Statistical analyses were performed in IBM SPSS 25 and 26.

## Results

116 patients underwent RC and SNd (fig) and of these, 83 patients received 1–4 cycles of NAC and were stratified by pathoanatomical outcomes, into *complete response* (*CR*), (pT0N0M0), *stable disease (SD)* (pTis-pT4aN0M0) and *progressive disease (PD)* pTanyN+ and/or pM+ (Table 1).

**Table 1:** Patient characteristics

	NAC					No NAC	All
	All NAC	CR—complete response	SD—stable disease	PD—progressive disease	*p *value		
Designation of outome	–	pT0N0M0	pTis-T4aN0M0	pTanyN+		All pT-stages	–
No. of patients	83	36	35	12		33	116
Age (mean)		67	66.8	69.3	0.61	75.8	69.7
Age (range)	39–80	39–79	39–79	58–80		57–87	39–87
Gender					0.91		
Male	66 (79.5)	28 (77.8)	28 (80)	10 (83.3)		19 (57.6)	85 (73.3)
Female	17 (20.5)	8 (22.2)	7 (20)	2 (16.7)		14 (42.4)	31 (26.7)
Clinical stage					0.04		
cT2	63 (75.9)	32 (88.9)	24 (68.6)	7 (58.3)		23 (69.7)	86 (74.1)
cT3	17 (20.5)	4 (11.1)	8 (22.9)	5 (41.7)		10 (30.3)	27 (23.3)
cT4a	3 (3.6)	0	3 (8.6)	0		0	3 (2.6)
No. of NAC-cycles					0.86		
1	6 (7.2)	3 (8.3)	2 (5.7)	1 (8.3)			
2	9 (10.8)	1 (2.8)	6 (17.1)	2 (16.7)			
3	62 (74.7)	30 (83.3)	24 (68.6)	8 (66.7)			
4	6 (7.2)	2 (5.6)	3 (8.6)	1 (8.3)			
NAC-type					0.89		
MVAC	25 (30.1)	11 (30.5)	11 (31.4)	3 (25)			
HD-MVAC	53 (63.9)	24 (66.7)	21 (60)	8 (66.7)			
Cisplatin-gemzar	4 (4.8)	1 (2.8)	2 (5.7)	1 (8.3)			
Carboplatin-gemzar	1 (1.2)	0	1 (2.9)	0			

Baseline characteristics for all 116 cystectomized patients distributed over subgroups. Statistical analysis was applied on NAC-patients only. There were no statistical differences between NAC-subgroups in age, gender, number of NAC-cycles or NAC-type. NAC-subgroups differed significantly in clinical tumour stage pre-RC (*p* = 0.04)

*NAC *neoadjuvant chemotherapy, *RC *radical cystectomy, *HD-MVAC *high dose Methorexate, Vinblastine, Adriamycin, Cisplatin

In the NAC-treated cohort, clinicopathological factors were compared between the subgroups. There were no statistical differences in age, gender or number of NAC cycles (Table 1). The pathoanatomical outcomes in the NAC-treated cohort (*n* = 83), were CR in 43.4% (36/83), SD in 42.2% (35/83) and PD in 14.4% (12/83) (Table 2). NAC-subgroups differed significantly in clinical tumour stage pre-RC, with a higher number of cT3 and cT4 tumours in SD and PD-patients (*p* = 0.04). Interestingly, there were significant differences in mean and median number of SNs per patient in CR and SD compared to PD (*p* = 0.034). The SN-detection rate was 91.7% in CR-patients and 58.3% in PD-patients (Table 3). There were higher FP-detection rates in CR-patients (36.1%), compared to the overall FP-detection rate of 29.3% (*p* = 0.36) (Table 3). However, in a multivariate linear regression model, the only significant predictor for SNs was the number of harvested nodes (Table 4).

**Table 2:** Pathoanatomical outcomes

Final pTNM	NAC			No NAC	All
	CR—complete response	SD—stable disease	PD—progressive disease		
pT0N0M0	**36**	0	0	**6**	**42**
pTisN0M0	0	**5**	0	0	**5**
pTaN0M0	0	**2**	0	0	**2**
pT1N0M0	0	**5**	0	**1**	**6**
pT2N0M0	0	**11**	0	**4**	**15**
pT3N0M0	0	**10**	0	**10**	**20**
pT4aN0M0	0	**2**	0	**1**	**3**
pT0N+	0	0	**1**	0	**1**
pTisN+	0	0	**1**	0	**1**
pT2N+	0	0	**4**	0	**4**
pT3N+	0	0	**5**	**7**	**12**
pT4aN+	0	0	**1**	**2**	**3**
pT4bN+	0	0	0	**1**	**1**
Any M+	0	0	0	**1**	**1**

Final pTNM-stages post-cystectomy for all included patients, stratified by subgroups. In the NAC-treated cohort; Complete Response (CR) was found in 43.4% (36/83), Stable Disease in 42.2% (35/83) and Progressive Disease in 14.4% (12/83) of the patients

**Table 3:** True positive and false positive sentinel node detections

	NAC					No NAC	All
	All NAC	CR—complete response	SD—stable disease	PD—progressive disease	*p *value		
Total no of harvested lymph nodes	1350	616	572	162		508	1858
Mean no of harvested lymph nodes	16.3	17.1	16.3	13.5	0.5	15.4	16
Sentinel nodes							
Total	262	120	125	17		102	364
Mean	3.2	3.3	3.6	1.4	**0.034**	3.1	3.1
Median	2	2.4	3	1	**0.049**	3	2.5
Rate of detection %	85.5	91.7	88.6	58.3		75.8	82.8
False positive nodes							
Total	42	25	13	4		18	60
Mean	0.51	0.69	0.37	0.33	0.36	0.55	0.52
Rate of detection %	30.1	36.1	22.9	33.3		27.3	29.3

Total and mean number of harvested lymph nodes, true sentinel nodes and false positive detections, for all cystectomized patients and by subgroups. A true positive detection was defined as a radioactive specimen with > 10 CPM confirmed as a lymph node by histopathology. Detections with CPM > 10 which did not contain any lymphatic tissue, were labelled as false positive (FP). There was a significant difference in both mean and median number of SNs between the NAC-subgroups (*p* = 0.034 and *p* = 0.049)

*CPM *counts per minute (measured by Geiger probe intraoperatively), *CR *complete response, *SD *stable disease, *PD *progressive disease, *NAC *neoadjuvant chemotherapy

**Table 4:** Factors impacting SN-yield

Predictors	True SNs
	Multivariate *p *value
Age	0.18
Gender	0.67
Total no harvested nodes	**0.0004**
No. of NAC-cycles	0.47

The total number of harvested lymph nodes was the only statistically significant predictor of SN yield

*RC* radical cystectomy, *NAC *neoadjuvant chemotherapy

## Discussion

In the present study, we saw that the mean and median number of sentinel nodes (SN) were significantly lower in patients with progressive disease. This is, to our knowledge, the first time an association between SN yield and pathological outcome in NAC-treated MIBC, is recorded.

The finding could be explained by previous observations, namely that metastatic deposits appear to block lymph vessels or redirect the lymphatic flow resulting in a lowered rate of SN detection in patients with more advanced disease [11]. In addition, the biological role of the lymphatic system could be considered. These vessels are not passive venues for mechanical spread of cancer cells, but rather they play a major role in tumor immune responses [24, 25]. A recent experimental study showed that mice with ablated lymphatics exhibited reduced intra-tumoral accumulation of cytotoxic T cells and increased tumor PD-L1 expression, causing rapid tumor growth. Additionally, impaired function of the peritumoral lymphatic vessels resulted in decreased migration of dendritic cells to draining SNs compared with normal flank skin-draining lymph nodes [26].

Therefore, we hypothesize that the condition of the lymphatics might be reflected in the SN status. Conversely, a deficient lymphatic system could imply a state of immunodeficiency, which can result in reduced responsiveness to chemotherapy [21]. Thus, *the number of SNs* could hypothetically be a surrogate marker for antitumoral immunological activity, and perhaps, responsiveness to NAC.

Nevertheless, the association between the number of SNs and pathoanatomic outcomes must be interpreted with caution. For instance, the only factor that impacted the yield of SNs in our multivariate analysis was the total number of harvested lymph nodes. Several limitations of the study must be taken into consideration: First, the study is a retrospective analysis of a prospective cohort, meaning that the material was stratified and analyzed according to post-hoc constructed groups. Second, there were many centers with relatively few patients per center. This runs the risk of introducing bias due to heterogeneity in terms of individual urologic surgeons and pathologists. For example, individual lymph node dissection practices could theoretically cause variations in the LND template, since the template was predefined but not explicitly controlled for. Third, the time between injection of radioactive tracer and performed SNd may have varied by hours between patients, this due to intraoperative difficulties or different surgical techniques. A prolonged operation, allows the tracer to increasingly disperse throughout the entire lymphatic drainage line, leading to a suboptimal SNd. Fourth, peritumoral injections of technetium comes with technical challenges, especially in cases of large localized tumours or tumours located in diverticulae. For the fifth, the CPM-registration can be difficult to interpret. In some cases, there would be one reading in the surgical field but another on the dissection table.

With the approval of check-point inhibitors in late-stage urinary bladder cancer, there is a need to find good predictive markers for successful immunotherapy. In the future, patients with less advanced and non-disseminated tumours will probably undergo check-point inhibition. The main precondition for successful check-point inhibition is the very presence of active anti-tumourally directed T effector cells. A significantly reduced amount of T effector cells might indicate less efficacy of that kind of immunotherapy. Hence, a high number of sentinel nodes may be a candidate marker of mounted and functional immune responses valuable for adjuvant immunological therapy.

## Conclusions and future perspectives

There was a significant difference in mean and median numbers of SNs after NAC, between patients with CR and SD compared to PD-patients, with a significantly lower number of SNs in patients with progressive disease. However, many factors impact the SN-yield. We hypothesize that the number of SNs might reflect the function of the regional lymphatic system, thus making SN-number a plausible surrogate marker for antitumoral immunological activity.
